# Physical and chemical characteristics of slag produced during Pb refining and the environmental risk associated with the storage of slag

**DOI:** 10.1007/s10653-020-00738-5

**Published:** 2020-10-13

**Authors:** Alicja Kicińska

**Affiliations:** Faculty of Geology, Geophysics and Environmental Protection, Department of Environmental Protection, AGH University of Science and Technology, Mickiewicza 30 av, 30-059 Kraków, Poland

**Keywords:** Metallurgical waste, Heavy metals, Environmental risk assessment, Geochemical factors and indices

## Abstract

Metallurgical waste originating from the Zn and Pb refining process presents varying chemical composition and physical properties and contains varying quantities of pollutants. In the study, both fresh and weathered samples of production slag from the final Pb refining process were used to determine its physical parameters and chemical composition as well as to perform an environmental risk assessment (RAC, mRAC) related to its storage. This paper focuses on determining parameters such as natural humidity (1.8%) and bulk density (1267 kg/m^3^) of the slag. Also, its toxicity was analysed using bio-tests, its pH was measured (11.92) and the content of sulphates (3.5 wt%), chlorides (0.3 wt%) and selected heavy metals (Cd, Cu, Fe, Mn, Zn, Pb) was determined. The Individual Contamination Factor was determined, yielding the following order of the hazard level: Pb > Cu > Zn > Cd > Fe > Mn. Based on the mobility of metals determined using the Mobility Factor, it was concluded that the hazard level followed the sequence Cd > Pb > Zn > Mn > Fe > Cu. The obtained water leaching results were compared to the values found in the legal regulations in force. Based on this comparison, it was established that the slag studied constitutes toxic waste containing considerable quantities of sulphides and chlorides. The material is also a considerable source of readily leaching elements (Cd, Zn and Pb), and thus any product created using the slag may become hazardous to the environment. Also, the waste studied must not be used in the form in which it is currently stored due to the leaching of particularly toxic elements (Cd and Pb) in water solutions with increasing acidity.

## Introduction

Metallurgical waste has varying and often quite complex chemical composition that is often hard to determine (Pearson et al. 2001). It also presents different physical properties and causes varying levels of contamination. The reason for this is its varying geological origin, different methods for obtaining raw materials as well as different processing technologies and production methods employed, which are frequently changed by introducing new additives, catalysts and other substances used to modify the properties of the final product (Acosta et al. 2001; Czop and Kajda-Szczesniak 2016; Kierczak and Pietrasik 2011). An example of such waste is slag. The properties of this type of waste produced in various metallurgical processes can vary greatly as it contains fluxing agents, metal oxides (also heavy metal oxides), impurities from metal ores (i.e. potentially toxic elements—PTEs) as well as the by-products of coal, coke or other fuel burning. The presence of PTEs in slags is associated with their chemical properties which manifest as amphoteric properties, their characteristic very low solubility at neutral pH and increased solubility of metals in acidic and alkaline soil environments (Baran and Antonkiewicz 2017; Carvalho et al. 2014; Tsirdis et al. 2012; Liu et al. 2018).

The majority of slag created in smelting processes (about 99%) is recycled and only 0.4% is disposed of (CSO 2019; Data on Poland 2019). Based on its origin, we can distinguish boiler slag, molten slag, granulated slag and blast furnace slag. Based on its chemical composition (classified according to the oxide modulus), slag is divided into: carbo-silicate slag, alumino-silicate slag and silicate slag. However, the most commonly used classification method is based on particle size and grades slag as fine, medium and coarse.

The preferred method of handling waste, both economically and environmentally, is its recycling by returning it to a closed process cycle or directing it to another production process as recycled raw material (as an additive in the production of ceramic construction materials or reinforced concrete, or as aggregate for construction of road embankments). However, for this to be possible in light of EU regulations, several requirements must be met (PN-EN 15167–1: 2007; PN-EN 206 + A1: 2016; PN-EN 13242 + A1: 2010). Most importantly the material must be safe for the soil and water environment as well as for living organisms, especially humans. The probability of the occurrence of any negative effect on living organisms caused by one or multiple stressors is verified in the environmental risk assessment process (Barrio-Para et al. 2017; Ettler et al. 2004; Håkanson 1980; Kicińska 2019a). This assessment can be conducted using a variety of statistical methods, expert review methods (based on the knowledge and experience of the expert) or based on chemical analysis methods, which are expensive and time-consuming but offer an empirical reflection of the actual condition of the environment and measurable determination of the physical and chemical properties of the waste studied.

In light of the above, a study of a selected group of waste, i.e. slag from the Pb refining process, was conducted with the aim to: (1) determine the major physical parameters of slag produced during the final processing of lead refining waste, (2) determine its chemical composition and (3) conduct an environmental risk assessment in relation to its potential storage based on the geochemical fractionation of metals using single-step and sequential chemical extractions.

The scientific novelty of this research involves a detailed geochemical description of slag produced during Pb refining, whose chemical composition and aging processes differ from those observed in typical blast furnace or granulated slag. Moreover, the research methods used allowed for assessing the impact of slags on the soil and aquatic environment, which may take place during their storage. The manuscript also evaluates the impact of weathering processes (metal leaching) that may occur when heaps are improperly secured (or security measures are lacking whatsoever) or when slags are inadequately used in the “closed process cycle”. The slags described in the manuscript are an incredibly difficult material to study, as evidenced by the large number of technological processed used in the investigations. However, they do deserve thorough examination as they offer numerous possibilities for recovering trace elements and, at the same time, necessitate minimisation of the impact of their storage on the environment.

## Study area

**Fig. 1:**
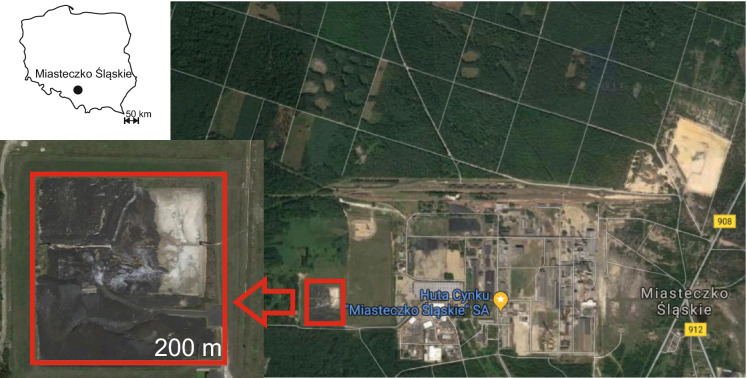
Location of slag dump in Miasteczko Śląskie

The site selected for the study is a zinc-works located in Miasteczko Śląskie (EU, Southern Poland, Silesia Province) which produces 80,000 tons of Zn (40% of Poland’s total output) and 6000 tons of Pb (50% of Poland’s total output) per year. The plant is Poland’s only manufacturer of rectified Zn and refined Pb and one of just eight such plants in the world. Production is conducted using the pyrometallurgical Imperial Smelting Process (ISP), involving both recycled material (i.e. materials containing Zn and Pb), comprising 80% of the feed and primary raw materials (mostly sulphide concentrates containing a blend of Zn and galena), comprising the remaining part of the feed for the shaft furnace. ISP is used in the Miasteczko Śląskie zinc-works due to its high efficiency and the possibility to process polymetallurgical raw materials. In the initial part of ISP, the feed is fully oxidised (sulphides are transformed into oxides) and the created zinc-lead agglomerate is transferred to the shaft furnace. After extracting gas from the furnace to the condenser, the separation process occurs. Zn is transferred to the refining process, and the liquid Pb is purified in a multi-step refining process. The aim of the refining is to purify raw Pb, getting rid of any admixtures and to recover accompanying metals (especially precious metals). Refining is also based on a pyrometallurgical process. In its final phase, saltpetre and caustic soda are used to separate Pb from slag which remains the only waste material produced by the zinc-works. About 700 tons of slag per year are transferred to the hazardous waste dump located in the western part of the plant (Fig. 1). The waste dump covers an area of 4.6 ha, and its total capacity is 350,000 tons. To date, 50,000 tons of the total capacity have been used. In the 1980s, this plant was considered one of the biggest industrial polluters in Poland as well as in Europe (8th largest polluter). Since the 1980s, numerous costly environment protection projects have been implemented to reduce the amount of pollution produced by the plant.

## Materials and methods

### Materials

Samples of fresh slag were collected directly from the process line in two lots, 2 weeks apart (in September 2018). The weight of each lot was about 30 kg. Then, 10 samples weighing 1 kg each were separated from each lot to obtain sample material with a total weight of 20 kg (PN-EN 14899: 2006). Next, the sample material was taken to the laboratory where it was sealed in PCV bags and put in a place with limited light and humidity. Before testing, the sample material was reduced (by quartering), homogenised and averaged, to obtain final samples in the form of 30 batches weighing 100 g each. The samples were dried at a temperature of 105 °C for 2 h and then placed in a desiccator.

### Analytical methods

In order to identify the physical properties of slag and its chemical composition, the following were determined: total humidity (PN-EN ISO 18134), water extract pH (1:10 ratio between solid phase and solution) and bulk density (PN-EN 12176: 2004). A toxicity analysis (bio-test) was also performed using seeds of *Lepidium sativum* L., and the phytoavailable quantity of selected elements was measured (0.02 M EDTA extraction using 1:10 solid phase/solution ratio). The use of *Lepidium Sativum* L. seeds as biological material serves as a fast and easy test for waste toxicity. It involves measuring the growth of roots in garden cress sprouts after 24 h of contact with waste and comparing the results with those obtained in a blank test (garden cress grown in distilled water). To determine the toxicity of the waste studied, 25 seeds were selected for each repetition of the test (the seeds had previously been prepared, i.e. they had been germinated, with the root length of approx. 1 mm). A layer of waste was placed in a Petri dish, moistened with distilled water and covered with filter paper. Then, about 25 garden cress seeds were placed in the dish. Next, the Petri dish was covered and placed in an incubator at approx. 25 °C for 24 h. The control sample was prepared in an analogical way—garden cress seeds were sown on filter paper only, which had previously been moistened with distilled water. After 24 h, the length of the roots was measured in the test and control samples. The experiment was repeated 20 times. The calculations were performed in accordance with the following formula:

1 $$I = \frac{{100\left( {L_{k} - L_{t} } \right)}}{{L_{k} }}$$

where *L*_*k*_— root length in the control sample (mean) [mm], *L*_*t*_—root length in the test sample (mean) [mm], *I*—inhibition [%].

Buffer properties and the metal release capabilities were tested using 7 acidity levels obtained by adding 65% nitric acid (in 7 doses from 0 to 2.34 mol). The quantity of the extracted Cd, Cu, Fe, Mn, Pb and Zn cations in the water extract was determined (PN-EN 12457–2: 2006) as was chloride (PN-ISO 9297) and sulphate content (PN-ISO 9280).

The chemical and phase composition of slag was determined using XRF and XRD instrumental analysis methods, respectively. The procedure used for both methods has been described in detail in Kicińska (2019a, b). In order to identify the morphology of mineral components in the slag samples studied, photographs of micro areas were taken using a scanning electron microscope (SEM) manufactured by FEI Quanta, model 200 FEG. The analyses were expanded by performing chemical tests of micro areas using an EDS detector. The tests were conducted in “high vacuum” mode. In order to improve the resolution of the microscope, samples were coated with carbon prior to the analysis. The accelerating voltage was 20 kV.

In order to conduct the environmental risk assessment (to calculate RAC and mRAC) related to the storage of slag, sequential BCR extraction was performed (Quevauviller 2003; Kicińska 2019c). The extraction performed allowed for establishing the percentage share of the following metal fractions: (1) exchangeable fraction—easily soluble in acidic environment. It contains metals found at the exchangeable positions or bound by carbonates, extracted with 0.1 M CH_3_COOH; (2) reducible fraction—susceptible to reduction. It contains metals bound by amorphous oxides/oxyhydroxides of Fe and Mn, extracted with 0.1 M NH_2_OH·HCl; (3) oxidisable fraction—susceptible to oxidation. It contains metals found in metaloorganic and sulphidic components, extracted with H_2_O_2_ and 1 M CH_3_COONH_4_; (4) residual fraction—elements contained in silicates, extracted with HCl:HNO_3_ (mixed at the 3:1 ratio).

The determination of elements present in the solutions was performed at the Accredited Hydro-Geo-Chemistry Laboratory at the AGH University of Science and Technology (certificate of accreditation PCA no. AB1050) using the inductively coupled plasma mass spectrometry (ICP-MS) method. The precision of element determination was 10% for Cd, Cu, Fe, Mn, Pb and Zn, while the accuracy varied between 95 and 105%, the DL of the appliance was 1·10^−5^ mg/dm^3^. Reference material used in the tests was CRM SQC001S from Sigma Aldrich (Lot No.: LRAB2028).

### Risk assessment indicators

Environmental risk (RAC) was calculated as a percentage share of the exchangeable fraction (step I) in the total content of a given element. The metal cations extracted in the first step of BCR occupy ion exchange positions or are bound to carbonates. Therefore, due to their relatively weak chemical bonds and easiness of migration, this fraction was deemed particularly harmful to the environment. It is readily absorbed by plants and thus can be easily incorporated in the trophic chain. If the calculated percentage is between 1 and 10%, the risk is low, between 11 and 30%, the risk is medium, between 31 and 50%, the risk is high, and if it is above 50%, the risk is very high (Pan et al. 2013; Kicińska 2019c). Also, the modified Risk Assessment Code (mRAC) was calculated based on formulas proposed by Håkanson (1980). Additionally, geochemical fractionation factors were calculated, i.e. the Mobility Factor (MF) and Individual Contamination Factor (ICF), which demonstrate the mobility of individual metals in relation to each other in the study material analysed (Håkanson 1980).

All calculations and statistical analyses were performed using the Statistica software ver. 13.1 and MS Excel. The Tukey’s HSD test (significance level of 0.05) was used to detect differences between means. Using statistical evaluation of the uncertainty of the parameters analysed, the quantity and type of material seem sufficient for proper geochemical reasoning. However, the research results obtained retain their exploratory character.

## Results

### Morphology and physical properties of slag

**Fig. 2:**
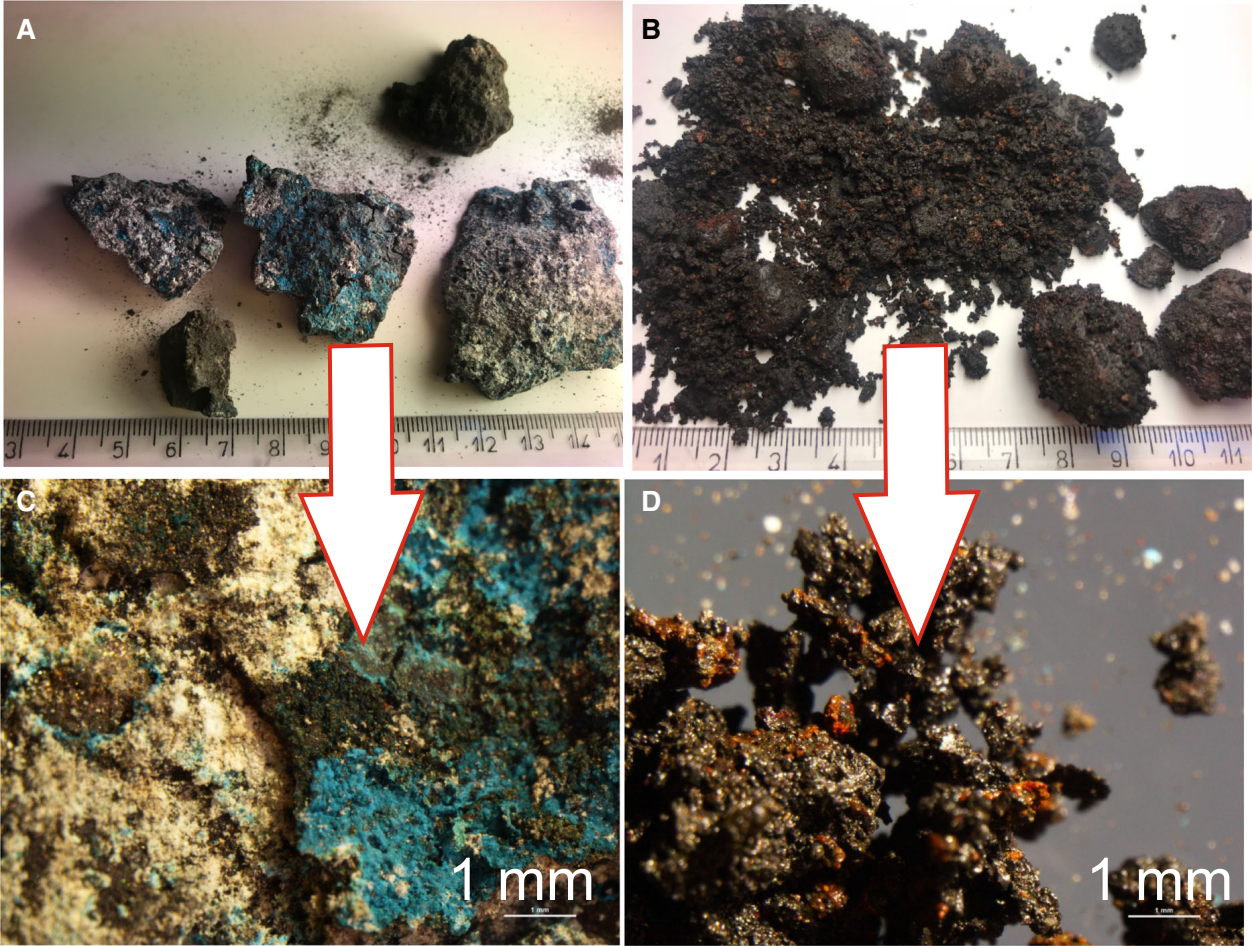
Macroscopic views of freshly produced (**a** and **c**) and weather-treated (**b** and **d**) slags

The material whose particle size, colour and physical properties were analysed was highly varied. Directly after collecting slag samples from the production line (i.e. fresh slag samples), their colour was dark, almost black. They also left coloured marks when rubbed against a coarse surface. When it comes to cohesion (consistence), the material was very hard, compact, non-crumbly and odourless. It had a high specific gravity as well as considerable porosity and a knobby structure. The dominant particle size was from 1.5 to 4 cm (Fig. 2a). The slag disintegrated to a large extent (Fig. 2b) after 4 months of seasoning (between September and December 2018) in normal weather conditions (temperate climate with transitional features), subject to: precipitation (about 100 mm in the September–December period), temperature (5*–*15 °C) and wind (north-western and western winds). Initially compact, lumpy and cohesive, the slag became crumbly and could almost be crushed in hands, leaving a strong coloured trace. Light-beige, rusty-red, and turquoise-blue efflorescence appeared on its larger pieces (about 1–2 cm) (Fig. 2c, d). Based on these observations, it was assumed that the material studied had been affected by rapid oxidation and hydration processes and that it contained up to several per cent of Na–Ca–K, Fe–Mn and Cu compounds. The instability of the forms of metals present in the slag was undoubtedly caused by the types of bonds formed during the primary processes and processing, and in the course of weathering, which must have been affected by changes of e.g. pH and Eh in the environment.

Due to the considerable variability of particle size in the material studied, the slag (fresh material) was divided into 3 groups for the purpose of microscopic description: non-sorted slag (NSS), coarse fraction slag (CFS) (≥ 10 mm) and fine fraction slag (FFS) (< 10 mm).

#### Non-sorted slag

**Fig. 3:**
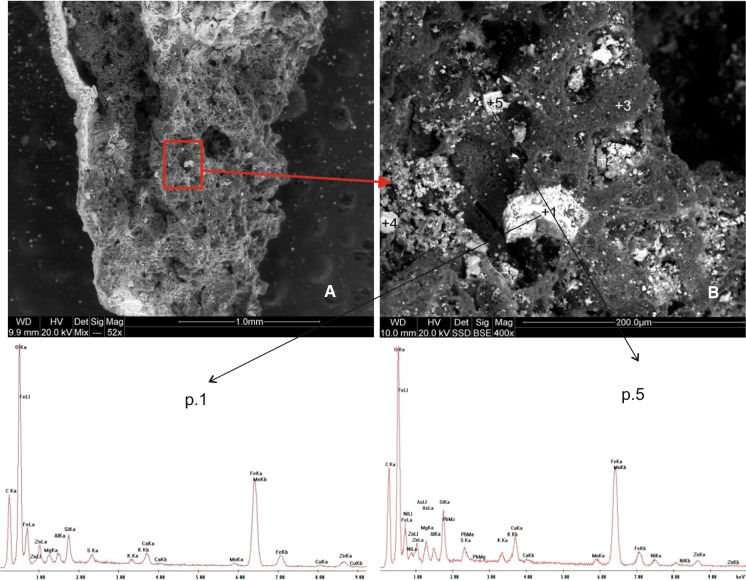
SEM images and EDS spectra of non-sorted fractions’ slag

The NSS group contained both large (about 3–4 cm) and smaller particles (< 1 mm). Their common feature was a very porous structure and uneven surface with smaller pieces of material attached to it (Fig. 3). They were of various shapes, from isometric angular forms (Fig. 3b, item 1 and 5), through elongated and irregular forms (Fig. 3b, item 2) to almost spherical forms (Fig. 3b, item 4).

Porosity of the slag structure is the result of a rapid release of gas in the cooling process, while the visible lighter grains are left by precipitated secondary mineral phases created in later phases of solidification. The spectra and the EDS-SEM element composition analysis indicated that these were mostly oxides, sulphides and rarely silicates (in wt%): Fe (5.8–27.9), Ca (0.7–11.4), Zn (0.5–2.5), Cu (0.3–1.4), Mn (0.6–1.0), Al (0.3–0.8), Mg (0.2–0.8), K (0.1–0.7), As (1.6), Pb (1.0) and Ni (1.6) (Table 1).

**Table 1:** Chemical composition (wt%) of slag from Miasteczko Śląskie Zn-works (NS)

Element	NS/BP1	NS/BP2	NS/BP3	NS/BP4	NS/BP5	Av. for all points
	(wt%)					
C	28.29	39.46	74.31	38.62	30.78	42.29
O	38.05	30.32	13.05	12.61	32.96	25.40
Mg	0.79	0.77	0.29	0.22	0.85	0.58
Al	0.79	0.66	0.53	0.30	0.81	0.62
Si	1.85	10.12	0.72	0.47	2.58	3.15
S	0.56	0.35	1.09	16.73	0.59	3.86
K	0.40	0.32	0.52	0.15	0.66	0.41
Ca	1.08	11.39	1.55	0.69	1.91	3.32
Mn	0.64	0.61	0.48	0.32	1.04	0.62
Fe	23.22	4.76	5.83	27.97	21.05	16.57
Zn	3.54	0.90	0.75	0.53	2.55	1.65
Cu	0.79	0.35	0.86	1.40	–	0.85
As	–	–	–	–	1.58	(1.58)
Pb	–	–	–	–	0.98	(0.98)
Ni	–	–	–	–	1.65	(1.65)
Total	100.000	100.000	100.000	100.000	100.000	99.32

*NS* Non-Sorted, –, not detected; P1–P5, point 1–5; see Fig. 5a (photograph B)

#### Coarse fraction slag

**Fig. 4:**
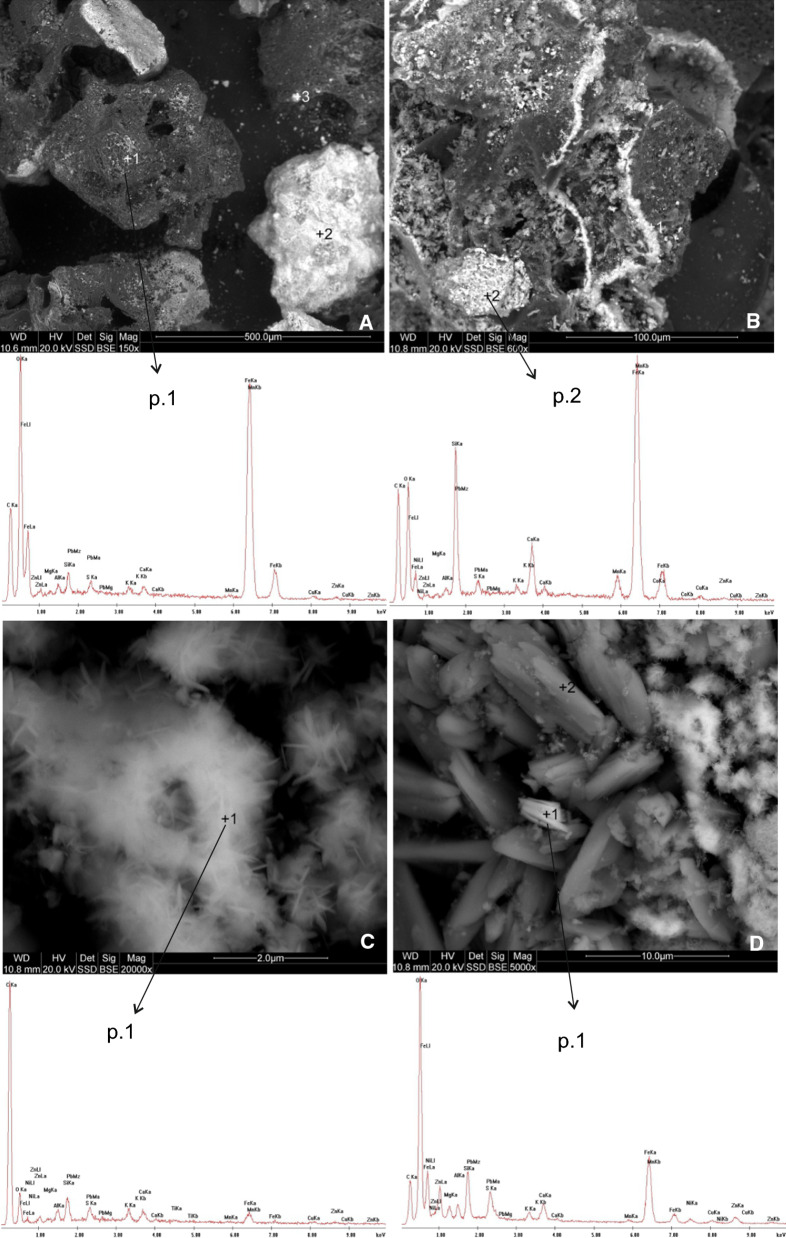
SEM images and EDS spectra of coarse fractions’ slag

CFS in the material analysed was characterised by large particles (1–5 cm) with an irregular surface, usually with sharp edges (Fig. 4a, b). Figure 4a shows four irregular particles. These are probably Fe carbonates, hydroxides and silicates with point-specific concentration ranging from 3.4 to 39.3 wt% (Table 2). Figure 4b shows a particle with a highly varied “sophisticated” structure and dimensions of about 200 × 300 µm. Numerous voids are filled with smaller forms (1–2 µm) which are presented in Fig. 4c and d. The fine lamellae, columns or needles sized between 1 and 10 µm are mostly composed of Ca and Fe carbonates. Their point-specific concentrations are 1.7–19.5 and 3.6–26.8 (wt%), respectively. The material studied also contained (≤ 1.6 wt%): Al, Mg, K, Mn, Zn, Cu, Pb, Ni, Ti, Co, Na and Mo (Table 2).

**Table 2:** Chemical composition (wt%) of slags from Miasteczko Śląskie Zn-works (CF)

Q`1	CF/AP1	CF/AP2	CF/AP3	CF/BP1	CF/BP2	CF/CP1	CF/DP1	CF/DP2	Av. for all points
	(wt%)								
C	74.55	19.91	26.98	29.93	31.75	24.29	29.83	32.14	33.67
O	11.45	41.54	27.06	38.84	13.87	41.69	28.43	40.49	30.42
Mg	0.25	1.06	0.27	0.59	0.17	0.62	0.55	1.34	0.61
Al	0.85	1.16	0.54	0.65	0.43	0.55	0.54	0.25	0.62
Si	1.59	3.45	0.93	1.69	6.08	0.72	1.48	0.38	2.04
S	0.95	1.43	0.46	0.49	0.59	–	0.48	0.23	0.66
K	1.22	0.70	0.46	0.44	0.46	0.24	0.38	0.20	0.51
Ca	1.24	1.73	0.57	0.59	2.68	1.73	10.42	19.52	4.81
Mn	0.44	0.67	0.51	0.23	2.92	–	0.59	0.10	0.78
Fe	3.46	17.92	39.26	22.39	37.94	26.79	19.67	3.61	21.38
Zn	1.38	4.21	1.23	1.08	1.05	0.78	2.55	0.55	1.60
Cu	1.44	1.63	1.22	1.11	1.31	1.03	1.76	0.85	1.29
Pb	0.75	2.96	0.51	1.71	0.23	–	2.02	–	1.36
Ni	–	1.66	–	0.28	0.20	–	–	–	0.71
Na	–	–	–	–	–	0.54	1.28	0.36	0.73
Ti	0.44	–	–	–	–	–	–	–	(0.44)
Co	–	–	–	–	0.31	–	–	–	0.31
Mo	–	–	–	–	–	1.05	–	–	(1.05)
Total	100.000	100.000	100.000	100.000	100.000	100.000	100.000	100.000	–

*CF* Coarse Fraction, –, not detected; P1–P3, point 1–3; see Fig. X, A–D no of photograph in Fig. 6a

#### Fine fraction slag

**Fig. 5:**
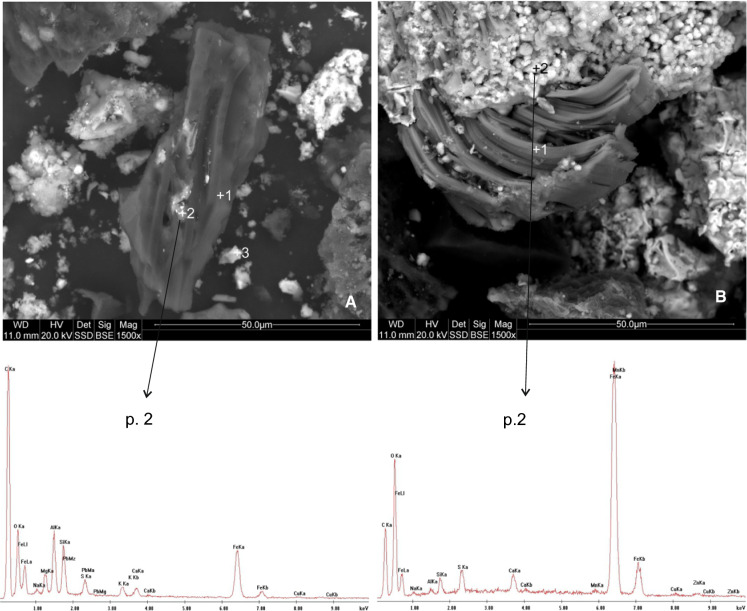
SEM images and EDS spectra of fine fractions’ slag

FFS contained considerably more of the finest material (< 5 µm). Particles with a very fine, often compact structure formed conglomerates or attached individually to slightly larger particles (Fig. 5a, b). In the central part of Fig. 5a there is an elongated 15 × 50 µm particle. This is probably a partially burnt piece of coal or quick coke whose surface is covered in very fine particles (about 1–5 µm) of silicates or Fe oxides. Figure 5b shows a crested gypsum structure, covered with conglomerated particles of Fe oxides and hydroxides. Similarly to NSS and CFS, the following elements were found in the material: Mg, Al, K, Ca, Mn, Zn, Cu, Pb and Na (Table 3).

**Table 3:** Chemical composition (wt%) of slag from Miasteczko Śląskie Zn-works (FF)

Element	FF/AP1	FF/AP2	FF/AP3	FF/BP1	FF/BP2	Av. for all points
	(wt%)					
C	87.96	62.80	47.92	28.54	24.69	50.38
O	8.44	15.49	8.05	36.91	17.92	17.36
Mg	0.14	1.25	0.10	–	–	0.50
Al	0.14	3.36	0.23	0.42	0.45	0.92
Si	0.32	2.34	1.20	0.49	0.92	1.05
S	0.45	0.77	11.64	11.88	1.26	5.20
K	0.11	0.70	0.20	–	–	0.34
Ca	0.32	0.70	0.45	13.95	1.34	3.35
Mn	–	–	0.93	–	0.84	0.89
Fe	1.55	11.17	28.03	6.28	49.50	19.31
Zn	–	–	–	0.66	1.59	1.13
Cu	0.30	0.56	0.99	0.51	0.93	0.66
Pb	0.06	0.51	–	–	–	0.29
Na	0.21	0.36	0.26	0.36	0.55	0.35
Total	100.000	100.000	100.000	100.000	100.000	–

*FF* Fine Fraction, –, not detected; P1–P3, point 1–3; A–B no of photograph in Fig. 7a

#### Other selected physical properties of slag

The measured total humidity of averaged (fresh) slag samples was 1.8 ± 0.2%. As this value is ≤ 2%, the material may be considered dry. It comes from high-temperature pyrometallurgical processes and since the temperature during those processes considerably exceeds 1000 °C, only scarce amounts of physically and chemically bound water are found in such material. The bulk density of slag is 1267 kg/m^3^. When classified in accordance with the PN-EN 13055 standard, the material studied has the properties of lightweight aggregate. The mean pH of slag (*n* = 30) measured in a water solution was 11.92 ± 1.21, which means it is strongly alkaline.

### Chemical and phase properties of slag samples

#### Total chemical composition (XRF) and phase composition (XRD)

The total detected chemical composition of slag (99.67 wt%) was divided into:

- *Major components*  ≥ 5 wt%. Their total content amounts to 83.52 wt% and they include Fe, Si, S and Ca oxides;
- *Minor components*  ≥ 1 wt%. Their total content amounts to 12.97 wt% and they include Al, Zn, Mn, Mg Na and K oxides;
- *Trace components*  < 1 wt%. Their total content amounts to 3.18 wt% and they include Pb, Cu, As, Ti, P, Ni, Ba, Co, Sr, Sb, Cr, Ga, Cr, Ga, Mo, V, Sn, Se, Zr, Ag, Ge, Rb, F and Cl oxides (Table 4).

**Table 4:** Components of slag origin from refined process

Component		Content per:		DL
		Component	Element	
		(% wt.)		
Main	Fe_2_O_3_	51.1132	35.7408	0.01114
	SiO_2_	12.4661	5.8286	0.00760
	SO_3_	10.3541	4.1494	0.00645
	CaO	9.5912	6.8557	0.00465
Sum (s_1_)		83.5246	52.5745	–
Secondary	Al_2_O_3_	3.9551	2.0939	0.00679
	ZnO	3.4562	2.7768	0.00396
	MnO	1.7328	1.3418	0.00632
	MgO	1.5200	0.9165	0.01355
	Na_2_O	1.2049	0.8940	0.00252
	K_2_O	1.0980	0.9115	0.00290
Sum (s_2_)		12.9670	8.9345	–
Trace	PbO	0.7934	0.7365	0.00731
	CuO	0.6387	0.5102	0.00431
	F	0.2540	0.2540	0.17344
	As_2_O_3_	0.2472	0.1872	0.02027
	TiO_2_	0.2221	0.1331	0.01059
	P_2_O_5_	0.2121	0.0926	0.00283
	NiO	0.1986	0.1561	0.00421
	BaO	0.1243	0.1113	0.02412
	Co_2_O_3_	0.0803	0.0571	0.00030
	SrO	0.0674	0.0570	0.00254
	Sb_2_O_3_	0.0662	0.0553	0.01727
	Cl	0.0540	0.0540	0.00656
	Cr_2_O_3_	0.0368	0.2518	0.00630
	Ga_2_O_3_	0.0352	0.0262	0.02284
	MoO_3_	0.0334	0.0223	0.00300
	V_2_O_5_	0.0274	0.0153	0.00138
	SnO_2_	0.0227	0.0179	0.01264
	SeO_2_	0.0210	0.0149	0.00366
	ZrO_2_	0.0117	0.0087	0.00295
	Ag_2_O	0.0181	0.0169	0.00649
	GeO_2_	0.0065	0.0045	0.00448
	Rb_2_O	0.0057	0.0052	0.00257
Sum (s_3_)		3.1768	2.7881	–
Total (s_1÷3_)		99.6684	64.2971	–

*DL* Detection Limit, –, not applicable

These data clearly indicate that in accordance with the oxide modulus the material studied belongs to ferruginous, silicate, carbon contain slags.

As for the share of elements in the slag, the dominant one was Fe, with a mean content as high as 35 wt% (Table 4). Ca, Si and S comprised 4–7%. The slag also contained considerable amounts of Zn, Al, Mn, Mg, K and Na. Their content was slightly lower than that of the elements listed above, ranging from 1 to 3 wt%. Two other elements, Pb and Cu, comprised less than 1 wt%. Finally, very low quantities (< 0.5 wt%) of As, Ti, P, Ni, Ba, Co, Sr, Sb, Cr, Ga, Cr, Ga, Mo, V, Sn, Se, Zr, Ag, Ge, Rb, F and Cl were also found in the material.

Such varied elemental composition of the slag analysed clearly confirms the polymetallic character of Zn–Pb concentrates processed and the considerable content of additives (Al_2_O_3_, NaOH, CaCl_2_, etc.) introduced in the multi-step technological process. Such additives may have an impact on the final composition of waste, including slag.

**Fig. 6:**
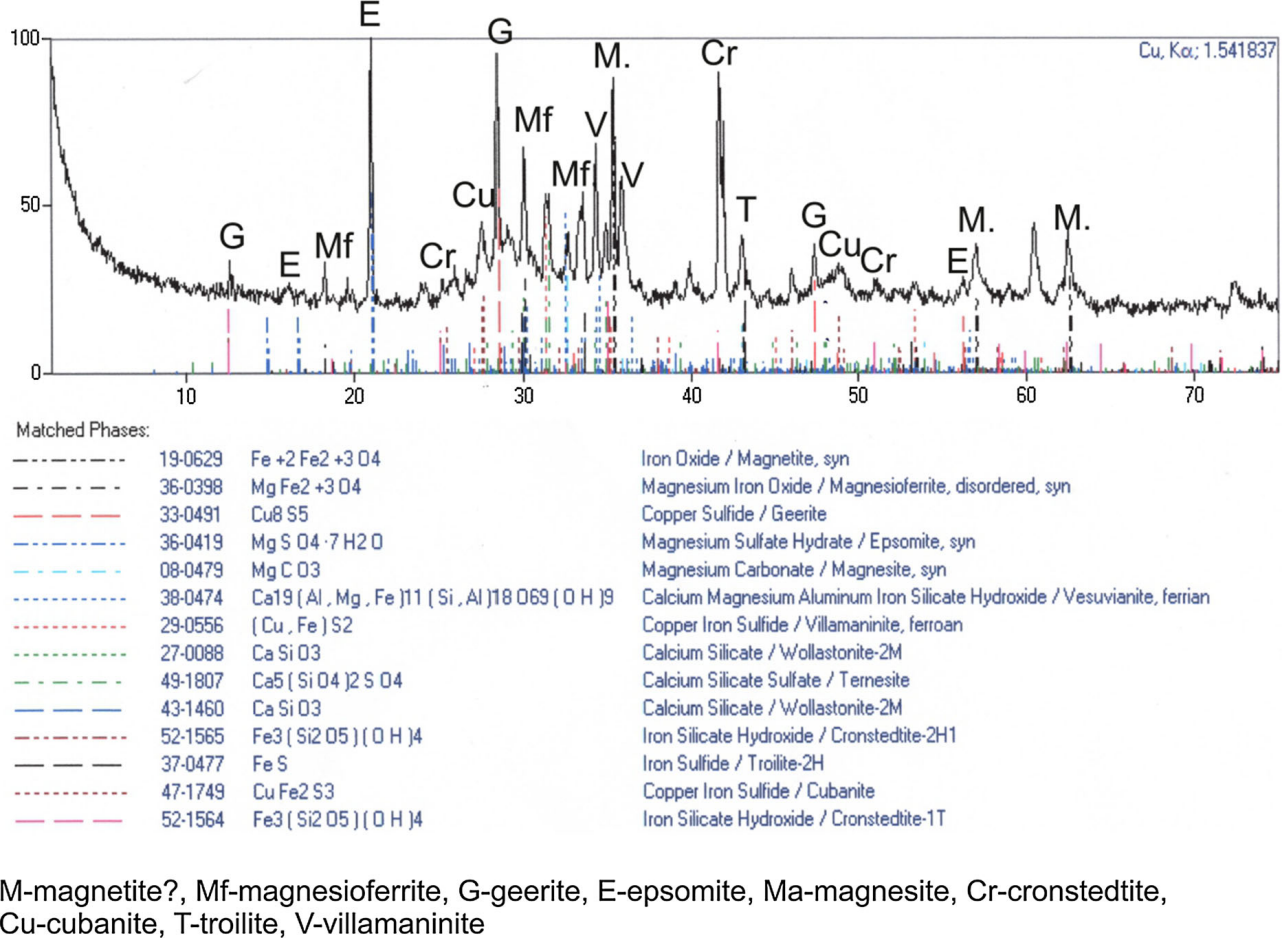
XRD patterns of slag sample

The diffractogram (Fig. 6) shows peaks caused by Cu and Fe oxides and sulphides, Ca and Mg sulphates, Mg, Ca and Fe hydroxides. Based on the spectrum recorded, magnetite, magnesioferrite, geerite, epsomite, vesuvianite, villamaninite, wollastonite, cronstedtite, troilite and cubanite were identified in the slag. Clearly, this is a peculiar combination of anhydrous and hydrous phases. It is undoubtedly caused by considerable hygroscopic properties of slag, which readily binds water from the environment (atmospheric air). Thus, the diffraction record shows hydrous mineral phases, i.e. epsomite and Fe–Si hydroxides, which would not normally be present in material subjected to high-temperature processing (at about 1200–1400 °C).

### Fractions binding PTEs in slags

**Fig. 7:**
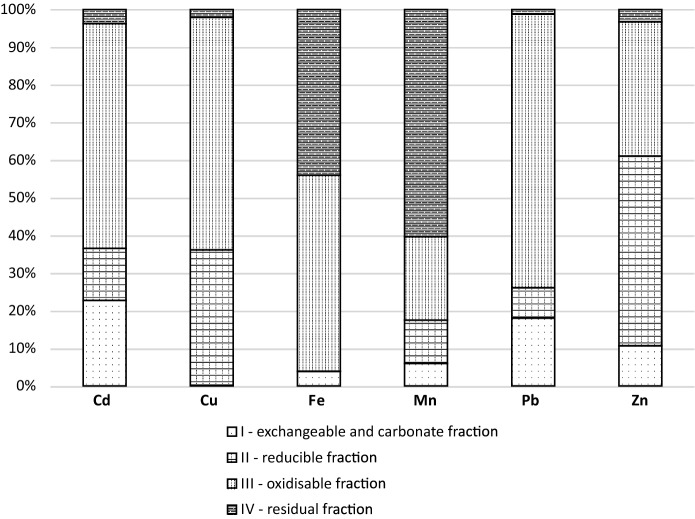
Share (%) of PTEs binding fraction in slag samples (mean, for *n* = 30)

Using environmental guidelines (Minister of Environment 2014) and based on studies related to the presence of PTEs (Cd, Cu, Fe, Mn, Pb, Zn) in the soil and plant environment published to date (Gbor et al. 2000; Yildrim and Prezzi 2011; Kicińska 2019b), the bound forms (geochemical fractions) of the above-mentioned elements were studied using four-step sequential BCR extraction (Fig. 7). The total quantities of the metals extracted during all 4 steps of the extraction were: Cd 6.6, Cu 942, Fe 108,870, Mn 7370, Pb 737 and Zn 4612 (mg/kg), respectively.

The following elements were in ion exchange positions and/or bound to carbonates, i.e. cations bound with weak chemical bonds (these quantities are presented as a percentage ratio between quantities determined in the first step and the total quantity determined in all four steps): Cu 0.18%, Fe 4%, Mn 6%, Zn 11%, Pb 18% and Cd 23%. The share of reducible forms (second step of BCR) of PTEs analysed was (in ascending order): Fe < Pb < Mn < Cd < Cu < Zn. Represented as percentage of the total amount determined during all four steps of the extraction, the results were as follows: 0.07, 8, 11, 14, 36 and 50 (%), respectively. The share of oxidised forms (third step of BCR) was considerably higher. In the case of Cd, Cu, Fe, and Pb it was over 50%, i.e. 60, 62, 52 and 73 (%), respectively. In the case of Mn and Zn, the quantity of cations bound in oxidised forms was 22 and 36 (%), respectively, of the total determined in all four steps of BCR extraction. After the fourth step of BCR less than 4% of Cd, Cu, Pb, and Zn were found in the residual fraction, while in the case of Fe and Mn the content was 44 and 60 (%), respectively.

### Leaching of PTEs from slags

#### Water leaching

Water leaching is a simple environmental test which allows for determining the amount of metals that may enter the water environment as a result of dissolution in a water solution. The water leaching test demonstrated that the following amounts of elements were extracted: Cd 0.0315, Cu 0.0005, Fe 0.3710, Mn 0.0008, Pb 0.0002 and Zn 0.0612 (mg/dm^3^), respectively (Table 5), were not exceeded. Thus, regarding the elements analysed, slag may be stored in an inert waste dump (Minister of Economy 2015). In the case of all metals analysed, the limit values established for the waste subject to the water leaching test were not exceeded. Similar conclusions apply to the conditions that have to be met when discharging sewage to water or soil, and substances particularly harmful to the aquatic environment. None of the limit values set for the parameters tested was exceeded (Minister of Environment 2014).

**Table 5:** Water leaching test (L/S 10:1) of PHE from slags

Element	Measured values		Limit value^1^ for waste deposited at landfills for			Permissible values of pollutant indicators for certain substances particularly harmful to the aquatic environment^2^	
			Inert waste	Non-hazardous and inert waste	Hazardous waste	Daily average	Monthly average
	mg/dm^3^	mg/kg	mg/dm^3^				
Cd	0.0315	0.324	0.04	1	5	0.4	0.2
Cu	0.0005	0.004	2	50	100	0.5	0.5
Fe	0.3710	2.367	–	–	–	10	10
Mn	0.0008	0.007	–	–	–	–	–
Pb	0.0002	0.001	0.5	10	50	0.5	0.5
Zn	0.0612	0.615	4	50	200	2	2

^1^According to the Regulation of the Minister for the Economy (2015)

^2^According to the Regulation of the Ministry of Environment (2014)

–, not establish

However, due to the results of the macroscopic analysis of the slag and the colourful efflorescence visible on its surface, it was decided to expand the water leaching analysis to include the content of Na, C and K. The following amounts of the elements were found in the water extract: Na 2339, Ca 3.6 and K 228 (mg/dm^3^). From the perspective of the aquatic environment, such a high content of Na may be a hazard to surface and ground water as the extracted quantity exceeds the safe limit (i.e. 800 mg/dm^3^). In the case of the remaining two elements (Ca and K), the regulation (Minister of Environment 2014) does not specify any limits for their release into the soil and water.

Also, the content of sulphates (SO_4_^2−^) in the water extract was determined at 3.5 wt%, which clearly indicates that this type of waste should be stored in a hazardous waste dump (Minister of Economy 2015). The high quantity of Na and sulphates in the slag is probably the result of sodium sulphide being used as flux in the technological process. Due to the high content of Na, the material may not be returned to the process or used in any other way, thus becoming a final waste that is transferred to a hazardous waste dump. The content of chlorides was 0.3 wt% and considerably exceeded the highest permissible value (1000 mgCl/dm^3^) for substances particularly harmful for the aquatic environment (Minister of Environment 2014).

#### EDTA leaching

The aim of extraction using EDTA 0.02 M solution was to determine the phytoavailable quantity of metals contained in the slag. Based on the extraction, the following quantities were found in the material: Cd 5.27, Cu 923.22, Fe 5 117.11, Mn 145.34, Pb 834.56, Zn 1571.22 (mg/kg). Kabata-Pendias and Pendias (1999) state that hazardous or toxic quantities for plants range between: Cd 3–30, Cu 800, Mn 400–1000, Pb 30–300 and Zn 100–400 (mg/kg). Therefore, the slag studied may be a hazard to plants due to the presence of high quantities of phytoavailable forms of Cd, Cu, Pb and Zn.

**Fig. 8:**
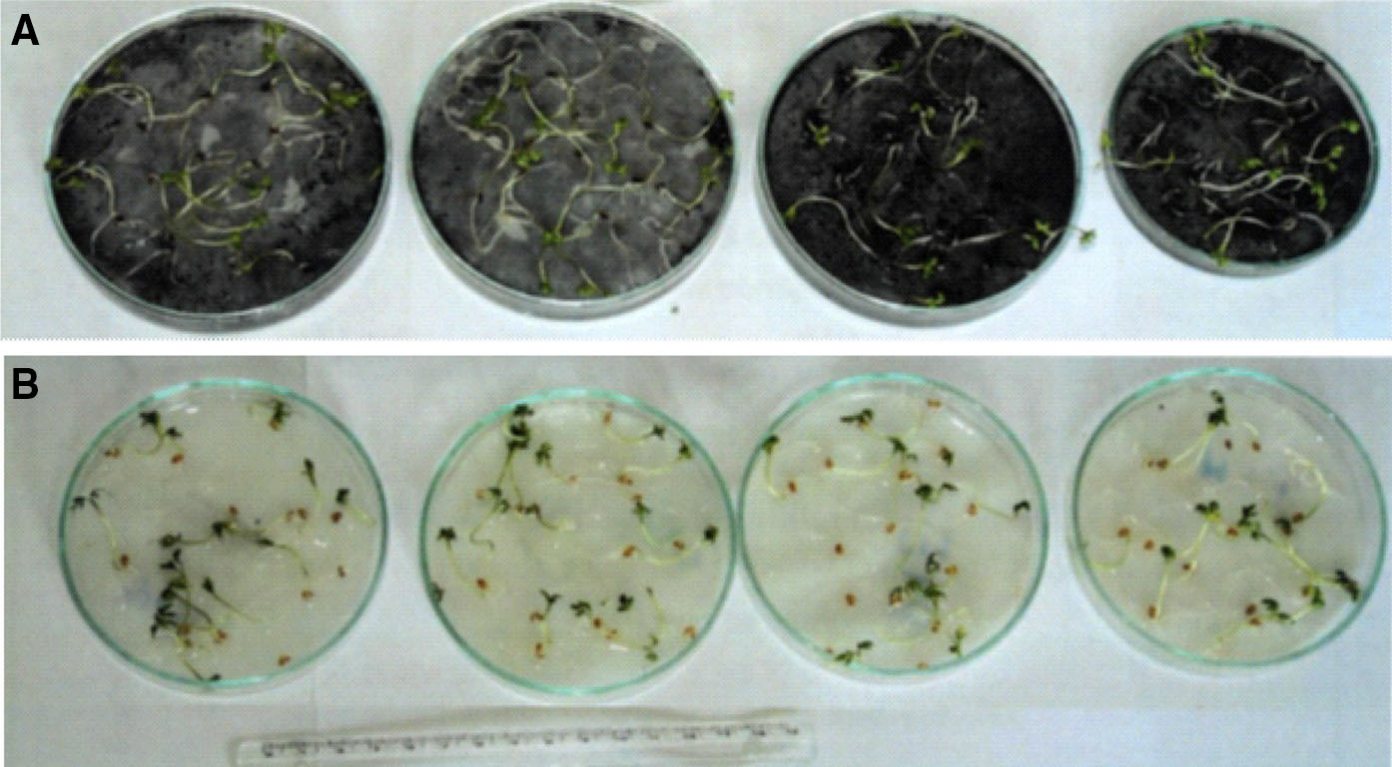
Biotest with cress seeds use. **a** a substrate with slag, **b** slag-free substrate—control sample

A bio-test using the seeds of *Lepidium sativum* L. (Fig. 8) was performed as a complementary analysis to the study on the impact of slag on living organisms. The test demonstrated that the growth of garden cress on a substrate containing slag was low, as opposed to the control sample (without the addition of slag). In 20 growth repetitions using a substrate containing slag, the length of roots that sprouted from the garden cress seeds was measured and the calculated growth inhibition factor I amounted to 90%. This result denotes that the waste studied is highly toxic to plants.

#### Acid leaching

**Fig. 9:**
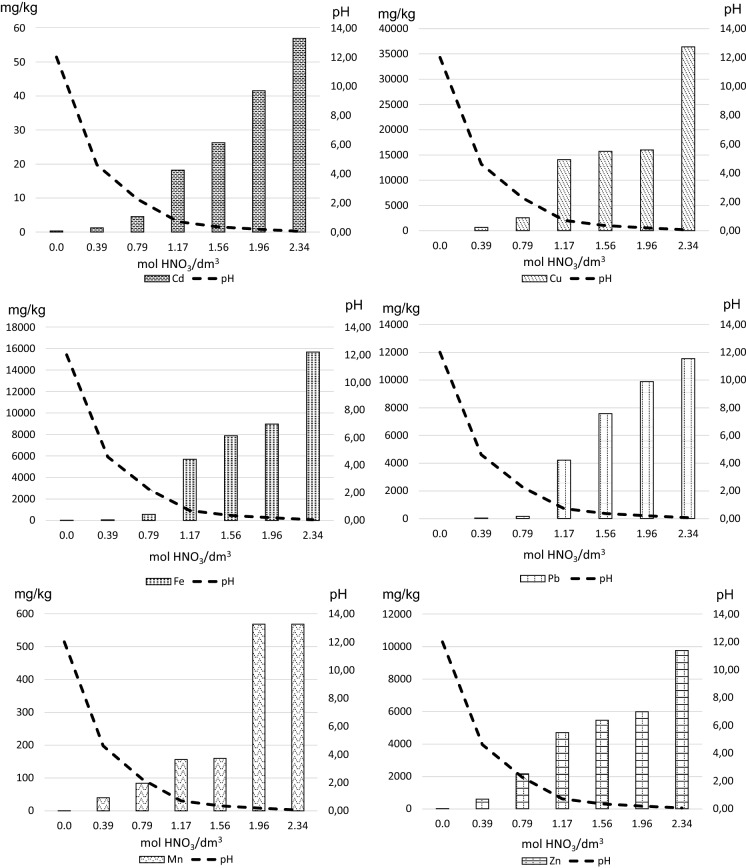
Metal leaching from slag due to acidification process

Another test determining the mobility of metals contained in the slag analysed involved acidification of solutions using 7 increasing doses of nitric acid (from 0 to 2.34 mol HNO_3_/dm^3^) and simultaneous measurement of pH (which allowed for determining the buffering capacity of the slag) and detection of the quantity of metal cations extracted by subsequent doses of acid (Fig. 9). The very first dose of acid (0.39 mol HNO_3_/dm^3^) caused a decrease in pH by 7.4 and the release of Cd 1, Cu 569, Fe 57, Mn 40, Pb 26 and Zn 587 (in mg/kg dry mass). Despite this considerable decrease in pH, it is important to note a very good buffering capacity of slags, as in theory the addition of 0.4 mol HNO_3_/dm^3^ should have caused a decrease in pH to a value close to zero. This may stem from the presence of high quantity of Ca and Mg compounds which were found in the material studied. Adding further doses of acid caused a decrease in pH by further 2 units, until the acidity reached pH 0.05 after the seventh dose. An increase in the quantity of metals extracted in the solutions was observed in subsequent steps of acidification. In the case of Cd and Zn, this increase was quite even, while in the case of Fe and Pb—it was small during the 3 initial steps of extraction and followed by a dynamic increase in the quantity of extracted Fe and Pb cations in further steps (Fig. 9). In the case of Mn, a small increase in the quantity of the extracted cations (from 40 to 160 mg/kg) was observed in the initial steps of extraction, while in the two final steps (doses of 1.96 and 2.34 mol HNO_3_/dm^3^) an almost identical quantity of cations was released (569 mg/kg). The situation was completely different in the case of Cu. Even the addition of a considerable dose of acid (1.96 mol HNO_3_/dm^3^) caused only a slow release of this element (from 629 to 15,984 mg/kg). In the final step (greatest acidity), as much as 36,421 mgCu/kg was released. These large quantities of Cu originate from primary raw materials and we do not currently have a technology to recover them. In the case of the other metals, the highest acidity caused the release of the following quantities of elements: Cd 57, Fe 15,698, Mn 569, Pb 11,547 and Zn 9,758 (mg/kg), respectively. Such large quantities of PTEs found in the solutions indicate that there exists a potential hazard to the soil and water environment caused by inadequate storage of slags and exposing them to acidifying substances (containing compounds such as SO_X_, CO and NO_X_).

### Environmental risk assessment (MR*, *ICF*,* RAC)

Estimation of environmental risk related to the over-normative presence of metals in various components of the environment may be conducted using numerous methods and based on various indices and factors (Håkanson 1980; Kicińska 2019c; Gong and Deng 2008; Chai et al. 2017). Popular indices and factors include MF, ICF and RAC, which use the results of geochemical fractionation based on sequential BCR extraction (Table 6).

**Table 6:** Geochemical indicators for the assessment of environmental risks associated with PHE content in slag

Element	MF	ICF	RAC	mRAC
Cd	22.7	25.5	Medium risk	Medium potential adverse of effect
Cu	0.2	48.2	No risk	
Fe	3.9	1.3	Low risk	
Mn	6.0	0.7	Low risk	
Pb	18.0	80.9	Medium risk	
Zn	10.7	29.9	Medium risk	

*MF* Mobility Factor, *ICF* Individual Contamination Factor, *RAC* Risk Assessment Code, *mRAC* modified Risk Assessment Code

The first of the calculated factors was the Mobility Factor (MF). The higher the factor’s value, the greater the mobility of the element in the environment. For the metals analysed, the following descending MF order was established: Cd (23) > Pb (18) > Zn (11) > Mn (6) > Fe (4) > Cu (0.2). The analysis of this sequence demonstrates that Cd, Pb and Zn are the most mobile elements. They are at the same time the most toxic to living organisms (Kabata-Pendias and Pendias 1999; Kicińska 2019a).

Another factor analysed was the Individual Contamination Factor (ICF), which describes the environmental risk. Similarly to MF, the greater the factor’s value, the higher the risk. The calculation of ICF values allowed for sequencing the elements analysed in the following descending order: Pb (81) >  > Cu (48) > Zn (30) > Cd (25) > Fe (1.3) > Mn (0.7). The high contents of Pb, Cu and Zn in slags stem from their content in concentrates processed at the metal-works. However, from the perspective of storing slags in a waste dump, their spreading and high toxicity may be a hazard to the health of living organisms as they accumulate the elements contained in the slags.

The Risk Assessment Code (RAC) analysis yielded the following results: No Risk in the case of Cu, Low Risk in the case of Fe and Mn and Medium Risk in the case of Cd, Pb and Zn. The total modified RAC (mRAC), taking into consideration the hazard caused by all elements analysed, indicated medium potential for adverse effect. This result was to be expected but at the same time it is not truly satisfactory.

## Discussion

Slag from metal-works is clearly not a material formed in natural processes. Slag has a complex chemical composition and varying physical properties. This has also been confirmed in the studies conducted by Gabor et al. (2000) as well as Yildrim and Prezzi (2011). Similarly, Ganne et al. (2006) found high concentrations of heavy metals bound to minerals such as arsenopyrite, franklinite and willemite in slag originating from Angleur (Eastern Belgium). Other authors demonstrated that in the short-, medium- and long-term (± 100 years) perspective, about 900 mg/kg Zn will be released to the soil and water environment as a result of natural acidification. Therefore, maintaining the correct environmental parameters such as pH is very important. The instability of metals in solids is caused by their form and changes of pH and Eh in the environment. An important feature of heavy metals is their very low solubility in neutral pH and increased solubility in an acidic environment. Each of them presents characteristic geochemical properties reflecting their individual dynamics of dissolution in acidic solutions and achieving maximum solubility at different pH values (Baran and Antonkiewicz 2017; Liu et al. 2018).

Elements detected in the slag occur in rather complex phases and chemical bonds, which is the result of high-temperature processing of ores and numerous transformations occurring at various stages of technological processes. The hydration process and the transformation of anhydrous forms into hydroxides is also the main cause of grain disintegration in slags and their increased crumbliness, as observed in the case of the weathered slag. The oxidation rate in the material studied, on the other hand, was confirmed by the presence of Fe sulphates (magnetite) which replaced Fe sulphides, i.e. marcasite (FeS) and pyrrhotite (FeS_2_).

The results of BCR extraction were quite predictable. Slags are not a natural matrix found in the environment, especially given that they are not produced in a natural process. They are a form of anthropogenic waste, subject to numerous transformations occurring in complex chemical and physical conditions. Therefore, the use of BCR extraction as a method for identifying bound forms/presence of metals in the case of slag remains disputable. However, it is the author’s opinion that such analysis may provide general information indicating the risk of release of some quantities of PTEs from various extraction solutions in a sequence of reagents with increasing aggressiveness.

As discussed by Morrison and Gulson (2007), heavy metals contained in slag may be harmful and even toxic to living organisms. In metal-works in Macquarie, New South Wales (Australia), there are 1.4 million tons of slag containing 2.5% of Pb and other pollutants. These researchers demonstrated that small particles containing large quantities of Pb, Cd and As (6490–41,400 ppm) may be swallowed by children and owing to the bioavailability of Pb, amounting on average to 45% in the case of ~ 250 μm particles, 75% in the case of smaller particles (53 ± 32 μm) and almost 100% in the case of the smallest particles (< 20 μm), they are a potential health hazard.

The toxicity and the level of environmental hazard are determined based on the concentration of the most dangerous component (including heavy metals), its toxicity and hazard posed to living organisms as demonstrated by the inhibition factor established in a bio-test. Inadequate storage and possible eolian deflation as well as washing out with rain water may be the source of considerable hazard to the environment.

However, it must also be remembered that there are potential uses for slag dumps. Estimations provided by Pozzi and Nowińska (2010) demonstrate that the hazardous waste dump in Miasteczko Śląskie where the slag studied is stored, contains vast amounts of elements, mostly metals, including (in Mg): Zn (4,620–8,000), Pb (6,161–16,500), Fe (1,540–20,900), Cd (880–3,190) and Cu (869–12,100).

## Conclusions

Based on the study conducted, it was found that:

1. The slag studied is a very heterogeneous type of waste. It is toxic and contains considerable quantities of heavy metals.
2. The chemical composition of the slag comprises primary components (Fe, Si, S and Ca), secondary components (Al, Zn, Mn, Mg, Na and K) and trace components (Pb, Cu, As, Ti, P, Ni, Ba, Co, Sr, Sb, Cr, Ga, Cr, Ga, Mo, V, Sn, Se, Zr, Ag, Ge, Rb, F and Cl).
3. The concentration of metals in different batches of slag may vary considerably and depend on the varying chemical and mineral composition of concentrates used as primary raw materials and processed in the metal-works.
4. The dominant mineral phases are Cu and Fe sulphides, Ca and Mg sulphates, Mg, Ca and Fe hydroxides.
5. Proper security of the waste dumps and heaps where the slag is stored is crucial as considerable amounts of metals as well as sulphates and chlorides may leach into the environment. The Mobility Factor indicated that the greatest hazard was caused by the presence of metals in the following order: Cd > Pb > Zn > Mn > Fe > Cu.
6. The waste studied must not be used in the form in which it is currently stored due to the leaching of elements that are particularly toxic (Cd, Pb and Zn) in water solutions with increasing acidity. Additionally, the slag should not be used in the production of e.g. concrete due to the high content of chlorides and sulphates.
7. Large quantities of the slag in waste dumps may become an anthropogenic source of elements to be used in the future. However, at present they require protection against leaching and washing out of the finest particles.
